# Development and performance evaluation of an artificial intelligence algorithm using cell-free DNA fragment distance for non-invasive prenatal testing (aiD-NIPT)

**DOI:** 10.3389/fgene.2022.999587

**Published:** 2022-11-29

**Authors:** Junnam Lee, Sae-Mi Lee, Jin Mo Ahn, Tae-Rim Lee, Wan Kim, Eun-Hae Cho, Chang-Seok Ki

**Affiliations:** ^1^ Genome Research Center, GC Genome, Yongin, South Korea; ^2^ Department of Bioinformatics, Soongsil University, Seoul, South Korea

**Keywords:** non-invasive prenatal testing (NIPT), cfDNA fragment distance, convolutional neural network (CNN), fetal chromosomal aneuploidy, artificial intelligence algorithm (AI)

## Abstract

With advances in next-generation sequencing technology, non-invasive prenatal testing (NIPT) has been widely implemented to detect fetal aneuploidies, including trisomy 21, 18, and 13 (T21, T18, and T13). Most NIPT methods use cell-free DNA (cfDNA) fragment count (FC) in maternal blood. In this study, we developed a novel NIPT method using cfDNA fragment distance (FD) and convolutional neural network-based artificial intelligence algorithm (aiD-NIPT). Four types of aiD-NIPT algorithm (mean, median, interquartile range, and its ensemble) were developed using 2,215 samples. In an analysis of 17,678 clinical samples, all algorithms showed >99.40% accuracy for T21/T18/T13, and the ensemble algorithm showed the best performance (sensitivity: 99.07%, positive predictive value (PPV): 88.43%); the FC-based conventional Z-score and normalized chromosomal value showed 98.15% sensitivity, with 40.77% and 36.81% PPV, respectively. In conclusion, FD-based aiD-NIPT was successfully developed, and it showed better performance than FC-based NIPT methods.

## Introduction

Since the discovery of cell-free fetal nucleic acids in maternal blood (Lo et al., 1997), non-invasive prenatal testing (NIPT) has been widely used to detect fetal chromosomal aneuploidy (Chiu et al., 2008; Chiu et al., 2010). Furthermore, with the advancement of next-generation sequencing (NGS) technology, new methods for the large-scale analysis of sequencing data have been developed.

Two sequencing strategies are commonly applied in NGS-based NIPT—namely, massive parallel sequencing (MPS) (Chiu et al., 2008), in which the entire chromosome is sequenced in low depth, and targeted sequencing (TS), in which only the target region of the chromosome is sequenced (Zimmermann et al., 2012; Pergament et al., 2014). In MPS, as the entire chromosome is sequenced, aneuploidy can be analyzed not only for chromosomes 21, 18, and 13 and for the sex chromosomes but also for other chromosomes (Pertile et al., 2017). In addition, maternal cancer can be screened *via* the analysis of the whole set of chromosomes (Bianchi et al., 2015). In TS, only a certain selected target region of the chromosome is amplified and sequenced; as the sequencing is selective, the cost is approximately 10-fold lower than that of the conventional MPS method (Sparks et al., 2012). Thus, TS can be used as a small bench-top sequencer; however, its application is limited as aneuploidy can be analyzed only for the target regions.

MPS is the main strategy applied in NGS-based NIPT, and various algorithms have been developed for bioinformatics analysis. Initially, the Z-score method was proposed (Chiu et al., 2008), followed by other methods, such as the normalized chromosomal value (NCV) wherein an optimized normalizing chromosome set is used on each target chromosome (Sehnert et al., 2011), an algorithm utilizing only the k-mer of the reads (NIPTmer) (Sauk et al., 2018), an algorithm that uses the cell-free DNA fragment length [COFFEE (Sun et al., 2017) and WisecondorFF (Mokveld et al., 2021)], a Bayesian statistics algorithm (Xu et al., 2018), and a graphic-aided algorithm (gNIPT) (Zhu et al., 2020). These algorithms analyze the count of the DNA fragment in a chromosomal region. As a count-based analysis, NIPT shows a sensitivity of 86.23%–99.02% and a positive predictive value (PPV) of 68.00%–85.27%, indicating a high level of technological performance; nonetheless, these results include the possibility of false positives and false negatives (Zhang et al., 2015; Zhu et al., 2020; Schmidt and Hildebrandt, 2021; Dar et al., 2022).

With the advancement of artificial intelligence (AI) algorithms, they have begun to be applied in genomic data analysis; e.g., the convolutional neural network (CNN) algorithm used to detect mutations (Poplin et al., 2018; Sahraeian et al., 2019). Other areas of application include gene prediction in the field of metagenomics (Al-Ajlan and El Allali, 2019) and motif finding in the field of epigenomics (Lanchantin et al., 2017), with ongoing developments of AI-based methods for various analytic purposes. For NIPT, the following algorithms were released: support vector machine, a machine learning algorithm to screen chromosomal aneuploidy (Yang et al., 2018), and a deep learning algorithm to estimate the fetal DNA fraction (Raman et al., 2019). Nevertheless, to our knowledge, no study thus far has reported on the use of deep learning in the screening of fetal chromosomal aneuploidy.

In this study, the concept of DNA fragment distance (FD) was applied for the first time, instead of the fragment count (FC)-based method for the analysis of fetal chromosomal aneuploidy. By using deep learning, four types of aiD-NIPT (AI using fragment distance-NIPT) algorithms were developed. Typically, in DNA FC-based analysis, only a single count value is used. In contrast, in analyses applying the concept of DNA FD, various representative values of the distribution, as well as an ensemble technique that combines such diverse values, can be used. The use of distance data with diverse values allows for a more accurate analysis than that when using a single value. In this study, using a deep learning algorithm, target repeat stacking (TRS) image generation was employed to train overall chromosomal patterns. This is what distinguishes our method from the conventional Z-test analysis, which cannot reflect the overall pattern of the chromosomes (Z-score and NCV score). The new method also includes the characteristic of learning and analyzing various features by the algorithms themselves. The performance of the newly developed algorithms was evaluated using a large-scale NIPT dataset against that of conventional Z-score and NCV-score analyses.

## Results

### aiD-NIPT model training result

In the development dataset, the accuracy of the test dataset was ≥99% across all models. The accuracy of each aiD-NIPT algorithm for trisomy 21, trisomy 18, and trisomy 13 was as follows: aiD_Ensemble, 99.92%; aiD_Interquartile range (IQR), 99.51%; aiD_Mean, 99.84%; and aiD_Median, 99.75% (Supplementary Table S2 and Supplementary Figure S1). The overall sensitivity was ≥95%, with the aiD_Ensemble and aiD_IQR showing the highest and lowest sensitivity, respectively. The aiD_IQR algorithm for trisomy 13 had a sensitivity of 80.00% (Supplementary Table S3).

**FIGURE 1:**
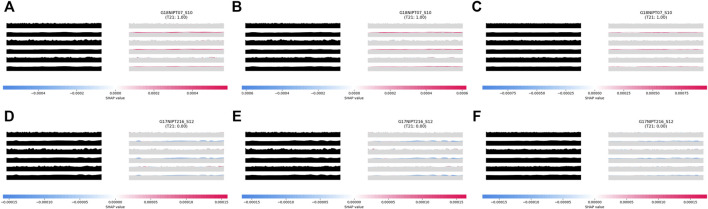
Distribution of feature importance for the aiD-NIPT algorithm for trisomy 21. The results of the aiD_IQR **(A)**, aiD_Mean **(B)**, and aiD_Median **(C)** analyses of the positive samples of trisomy 21, and the results of the aiD_IQR **(D)**, aiD_Mean **(E)**, and aiD_Median **(F)** analyses of the normal samples. The red SHapley Additive exPlanations (SHAP) value indicates positive, and the blue SHAP value indicates negative feature importance for the corresponding region. The feature importance on the edge of the TC at the 2nd, 4th, and 6th positions on the target repeat stacking image is shown to be high, suggesting that the model has learned to identify positive and negative samples by analyzing the overall pattern of TC. aiD: artificial intelligence of fragment distance; IQR: interquartile range; NIPT: non-invasive prenatal testing; TC: target chromosome.

The results of the trained model were verified through the SHapley Additive exPlanations (SHAP) value of the TRS image. A high value of feature importance could be seen on the ridge of the target chromosome at the 2nd, 4th, and 6th positions. This result suggests that the model using the TRS image learned the overall pattern of the target chromosome for the analysis (Figure 1).

### Comparison of the performance of each aiD-NIPT algorithm for the clinical dataset

The 17,678 clinical samples were obtained from pregnant women with a mean age of 35.58 years. The 25% and 75% percentiles of maternal age were 34 and 38 years, respectively. The percentage of those aged ≥35 years in the high-risk group was 67.38%. The mean gestational age was 14 weeks, and the percentage of the 12-week sample was the highest at 36.5%. The 11–13-week and 16–17-week samples accounted for 82.8% of all samples.

Algorithms using aiD_Ensemble and aiD_Mean showed the highest sensitivity (99.07%) for the clinical samples followed by aiD_IQR (98.15%) and aiD_Median (97.22%). Across the aiD-NIPT algorithms, except aiD_Median, trisomy 18 had one false-negative result. For the aiD_Median algorithm, trisomy 18 had two false-negative results. In the aiD_IQR and aiD_Median algorithms, trisomy 21 had one false-negative result. The aiD_Ensemble showed the highest PPV at 88.43%, followed by aiD_Mean at 87.70%, aiD_IQR at 80.92%, and aiD_Median at 67.74%. Comparison of the aiD_Ensemble and aiD_Mean algorithms showed that the aiD_Mean algorithm had one additional false-positive result.

Considering the sensitivity and PPV simultaneously, the aiD_Ensemble algorithm, utilizing all three FD representative values, showed the highest performance (Table 1). The aiD_Ensemble algorithm had a sensitivity of 100% and PPV of 96.59%, which was the highest for trisomy 21. The aiD_Mean algorithm had a PPV of 81.82%, which was the highest for trisomy 18, whereas all four algorithms showed the same sensitivity. The aiD_Ensemble and aiD_IQR algorithms had a sensitivity of 100% and PPV of 66.67%, which were the highest for trisomy 13 (Supplementary Table S4).

**TABLE 1:** Overall performance of each algorithm on the clinical dataset.

Overall model	True positive (n)	False negative (n)	False positive (n)	True negative (n)	Sensitivity (95% CI)	Specificity (95% CI)	PPV (95%CI)	NPV (95%CI)
Z-score	106	2	154	17,416	98.15 (95.61–100)	99.12 (98.99–99.26)	40.77 (34.8–46.74)	99.99 (99.97–100)
NCV score	106	2	182	17,388	98.15 (95.61–100)	98.96 (98.81–99.11)	36.81 (31.24–42.38)	99.99 (99.97–100)
aiD_Ensemble	107	1	14	17,556	99.07 (97.27–100)	99.92 (99.88–99.96)	88.43 (82.73–94.13)	99.99 (99.98–100)
aiD_IQR	106	2	25	17,545	98.15 (95.61–100)	99.86 (99.8–99.91)	80.92 (74.19–87.65)	99.99 (99.97–100)
aiD_Mean	107	1	15	17,555	99.07 (97.27–100)	99.91 (99.87–99.96)	87.7 (81.88–93.53)	99.99 (99.98–100)
aiD_Median	105	3	50	17,520	97.22 (94.12–100)	99.72 (99.64–99.79)	67.74 (60.38–75.1)	99.98 (99.96–100)

CI: confidence interval; PPV: positive predictive value; NCV: normalized chromosomal value; aiD: artificial intelligence of fragment distance; IQR: interquartile range.

### Comparison of the performance of aiD-NIPT and conventional algorithms for the clinical dataset

The performance of aiD-NIPT, a novel model developed in this study, was compared with that of the most well-known conventional NIPT algorithms (i.e., Z-score and NCV-score algorithms). Compared to the conventional NIPT algorithms that apply the mean and standard deviation (SD) of a reference set, the AI-based aiD-NIPT showed superior performance in terms of both sensitivity and PPV. A marked improvement in the PPV was observed for aiD_Ensemble, aiD_IQR, and aiD_Mean (>80%), as compared with those of the Z-score (40.77%) and NCV-score algorithms (36.81%) (Table 1).

The sensitivity for trisomy 21 was 98.82% using the Z-score and NCV-score algorithms; 100% using aiD_Ensemble and aiD_Mean; and 98.82% using aiD_Mean and aiD_Median. Among 85 samples confirmed for trisomy 21 by amniocentesis, one sample presented false-negative results in algorithms using Z-score and NCV, with a Z-score of 2.57 and NCV score of 2.46. However, the aiD_Ensemble, aiD_Mean, and aiD_Median algorithms showed a positive result with aiD_Ensemble at 0.87, aiD_Mean at 0.68, aiD_Median at 0.81, and aiD_IQR at 0.90.

## Discussion

In this study, a novel AI algorithm (aiD-NIPT) employing a distance-based concept for the detection of fetal chromosomal aneuploidy was developed and applied to an AI algorithm, the CNN. The diagnostic performance of aiD-NIPT was evaluated, and it was found to be superior to conventional NIPT algorithms.

**FIGURE 2:**
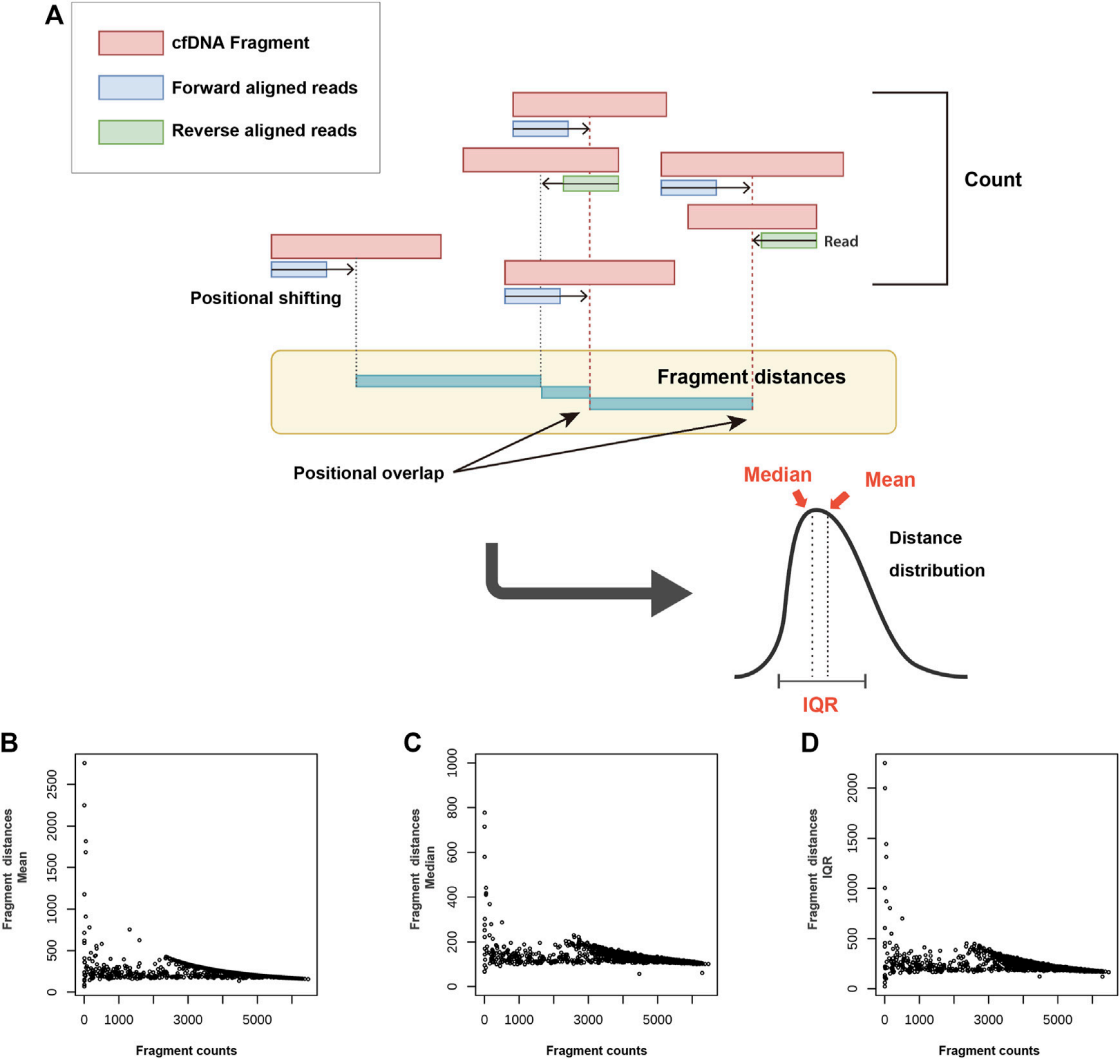
Concept and characteristics of fragment distance (FD) **(A)** and its relationship with fragment count (FC) **(B–D)**. After positional shifting in the direction of aligned reads, the distance between the closest positions is calculated. While the count is obtained as a single value based on a specific region, the FD allows the use of various representative values (mean, median, and IQR) through the distribution data. With respect to the relationship between the FC and FD representative values [mean **(B)**, median **(C)**, and IQR **(D)**], the FD is shown to decrease as the FC increases. This trend, however, is not observed across all regions. cfDNA: cell-free DNA; IQR: interquartile range.

The most important property of FD is that it can utilize distribution information. The several distance representative values calculated from the distribution can be used in various combinations (Figure 2A). The mean, median, and IQR were the FD representative values used in this study. With the exclusion of repeat regions, such as centromeres and telomeres, and following the non-overlapping binning at 1 Mb, the FC and FD (mean, median, and IQR) values were compared for each bin. As the FC increased, a decrease in the FD was observed. However, the number of regions exhibiting the same FD, but a different FC value was significant. A characteristic feature of unclear inference of the FD from FC was observed across all FD representative values (i.e., mean, median, and IQR) (Figures 2B–D).

The algorithms using mean, median, and IQR and the ensemble algorithm combining all three representative values were compared. The ensemble algorithm showed the best performance. The highest overall model sensitivity (99.07%) was observed for aiD_Ensemble and aiD_Mean; aiD_Ensemble showed the highest PPV at 88.43%. The most important performance value for the NIPT analysis was sensitivity. The aiD_Ensemble algorithm had the highest sensitivity (99.07%), while also having the highest PPV (88.43%). This indicates the advantages of FD, which allows the use of various representative values.

The performance of the distance-based AI analysis was better than that of the count-based statistical Z-test algorithm. Across a total of 85 samples confirmed on trisomy 21, one sample showed a false-negative result below the cutoff, with a Z-score of 2.57 and NCV score of 2.46; in contrast, this sample showed a positive result with aiD_Ensemble at 0.87, aiD_Mean at 0.68, aiD_Median at 0.81, and aiD_IQR at 0.90. The comparison of the cutoff and calculated values for this sample revealed that the value obtained through the statistical Z-test algorithm (Z-score and NCV score) was close to the cutoff set at three, indicating the possibility of ambiguity between false-positive and false-negative results depending on the slight adjustment of the cutoff. In contrast, the values obtained through aiD-NIPT were ≥0.8, except for aiD_Mean. Compared with the cutoff set at 0.5, the possibility of false-positive and false-negative results was lower, suggesting higher robustness in positive detection.

**FIGURE 3:**
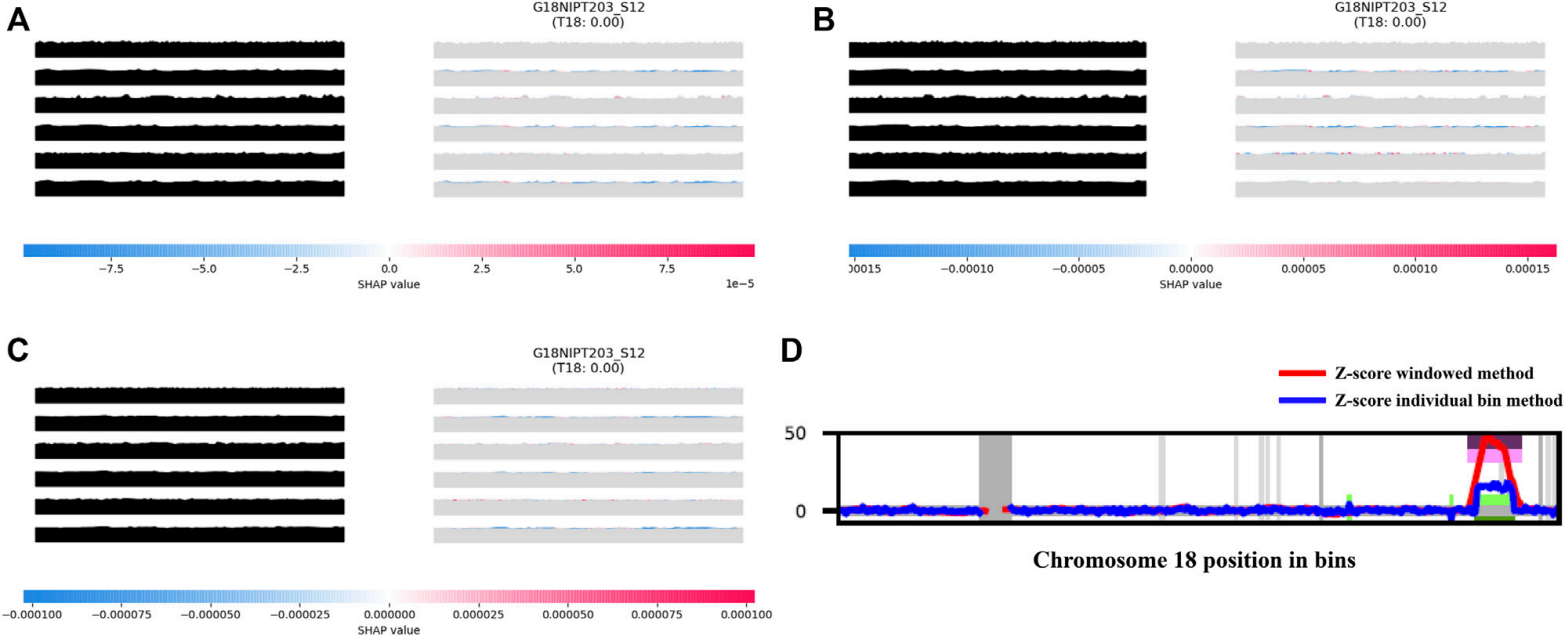
aiD-NIPT analysis; aiD_IQR **(A)**, aiD_Mean **(B)**, and aiD_Median **(C)** of the sample with trisomy 18 false-positive result in the conventional count-based analysis, and the result of the bin analysis **(D)**. The feature importance of the TC at the 2nd, 4th, and 6th positions on the target repeat stacking image displays a negative value on the distribution. Through the WIthin-SamplE COpy Number aberration DetectOR (WISECONDOR) analysis, the subchromosomal copy number variant on chromosome 18 was found. Such regions were removed in the aiD-NIPT training process. aiD: artificial intelligence of fragment distance; IQR: interquartile range; NIPT: non-invasive prenatal testing; SHAP: Shapley additive explanations; TC: target chromosome.

This result is associated with the characteristics of AI analyses. In the process of TRS image generation, the impact of maternal copy number variation (CNV) could be minimized while using the overall pattern of a chromosome rather than a single chromosomal value for analysis during model training. For instance, a sample from a 40-year-old pregnant woman was classified as positive in the Z-test analysis (Z-score of 8.77 and NCV score of 8.76), but then confirmed as negative. The aiD-NIPT result for this sample was negative across all models. The CNV analysis of this sample detected a large CNV of approximately 5 Mb from 68,000,000 to 73,600,000 on chromosome 18. The feature importance through the SHAP value showed that the CNV did not influence the aiD-NIPT analysis (Figure 3). In the presence of both copy number loss and gain on a single chromosome, the conventional method displayed the possibility of false-positive and false-negative results due to the influence of the size of the loss and gain regions and the maternal-fetal concordance. However, as the aiD-NIPT analysis uses the overall pattern of a chromosome in training, a more accurate analysis is possible.

In the learning of patterns and their analyses, it is not easy to select suitable features across a large and complex dataset. Selecting a feature with inadequate explanatory power can lead to the learning of noise and inaccurate results, whereas the underfit may prevent learning. The CNN used in this study is characterized by its ability to autonomously and simultaneously perform feature selection and classification for the given data. The autonomous process of feature selection allows a suitable feature to be selected for the data characteristics (Suriya et al., 2019). An algorithm with such an ability could be realized in NIPT, which showed superior performance compared with conventional methods.

One false-negative case was identified as trisomy 18, which was a low-risk result by all algorithms. This sample was from a 38-year-old pregnant woman at 12 + 4 weeks of gestational age. The calculated fetal fraction was 6.0 with a Z-score of −0.40 and an NCV score of 0.17; all aiD_Ensemble, aiD_IQR, aiD_Mean, and aiD_Median algorithms had a value of 0.00 (Supplementary Figure S2). The impact of maternal CNV seemed negligible, and the genotype of the placenta sample could not be evaluated. The sample was speculated to be a case of true fetal mosaicism for fetal chromosomal aneuploidy with normal placenta (Grati, 2016).

The PPV of the aiD_Ensemble algorithm on 17,678 samples was 96.59% for trisomy 21 and 66.67% for trisomy 18 and trisomy 13. The PPV was relatively lower for trisomy 18 and trisomy 13 owing to the small sample size of confirmed trisomy 18 (*n* = 19) and trisomy 13 (*n* = 4). The calculated PPV values, however, fell within the range reported in other studies. According to previous studies, the PPV is 68%–98% for trisomy 21, 47%–89% for trisomy 18, and 14%–83% for trisomy 13 (14–16), (Meck et al., 2015; Petersen et al., 2017; Yamada et al., 2018; Chen et al., 2019; Hu et al., 2019; Wan et al., 2021).

This study had some limitations. Although the model performance was compared on a large scale using 17,678 samples, the positive sample was small for trisomy 18 and trisomy 13 for accurate evaluation of performance. In addition, the algorithm validation on rare autosomal trisomy (RAT) and twin pregnancies were not possible due to the lack of positive samples. As studies have reported that analysis of RATs and multiple fetuses is possible using Z-score and NCV-score (Grömminger et al., 2014; Scott et al., 2018), the method used in this study can be applied if there are sufficient positive samples. Furthermore, the performance could not be evaluated for sex chromosomes and the association with the fetal fraction could not be verified despite the significant known impact on the NIPT results.

This study used the concept of FD for the first time and validated representative values (i.e., mean, median, and IQR) and an ensemble algorithm. As distribution information is available from the FD, various representative values other than those used in this study can also be utilized. The CNN algorithm for the AI-based analysis requires a considerably large number of learning parameters in order for the analysis to be conducted in a place equipped with adequate computational power. The identification of the optimal combination of key parameters, such as image size, number of convolutional layers, and patch size, is important. Although temporal and spatial factors are excluded from the features owing to the limitations of the CNN algorithm, a more promising model is possible through fusion with a recurrent neural network algorithm that overcomes such limitations.

A novel algorithm for detecting fetal chromosomal aneuploidy was developed based on the concept of DNA FD and an AI algorithm, and the algorithm performance was evaluated. The performance of this novel algorithm was better than that of conventional Z-score-based algorithms utilizing the mean and SD of a reference set. Recently, numerous studies have reported the use of low-coverage whole-genome sequencing data in early cancer diagnosis and minimal residual cancer detection. AI algorithms that apply the FD and TRS image generation similar to that in this study are expected to be useful in the future for applications in other fields.

## Methods

### Sample collection

Blood samples were collected from 20 to 45-year-old women with singleton pregnancy, and a total of 19,893 NIPT cases were used. The samples were divided into two groups: the development dataset (*n* = 2,215) for machine learning training and the clinical dataset (*n* = 17,678) for algorithm validation (Table 2). The clinical dataset consisted of the samples with invasive confirmation test (amniocentesis) results and samples with the results confirmed over the phone. This study was approved by the Institutional Review Board of Green Cross Laboratories, Yongin-si, Gyeonggi-do, Republic of Korea (IRB approval no: GCL-2021-1048-01).

**TABLE 2:** Composition of the development and clinical datasets.

	Development dataset		Clinical dataset	
	No. of samples	Percentage (%)	No. of samples	Percentage (%)
Gestational age				
1st trimester	833	37.61	9,296	52.59
2nd trimester	1,367	61.72	8,047	45.52
3rd trimester	4	0.18	23	0.13
Unknown	11	0.50	312	1.76
Maternal age (years)				
20–24	18	0.81	157	0.89
25–29	132	5.96	1,118	6.32
30–34	549	24.79	4,492	25.41
35–39	1174	53.00	9,637	54.51
40–45	342	15.44	2274	12.86
Sample type				
Low-risk	1,981	89.44	17,570	99.39
Trisomy 21	162	7.31	85	0.48
Trisomy 18	56	2.53	19	0.11
Trisomy 13	16	0.72	4	0.02
Fetal Fraction (%)				
0–10	611	27.58	5237	29.62
10–20	1546	69.80	12,147	68.71
20–30	58	2.62	287	1.62
>30	0	0.00	7	0.04

### Library preparation and sequencing

Approximately 10 ml of maternal blood in a Streck Cell-Free DNA BCT^®^ tube was centrifuged at 1,600 × *g* for 10 min and then for another 10 min at 3,000 × *g* to isolate the plasma. Cell-free DNA was extracted from 1 ml of the isolated plasma using the Tiangen micro DNA kit (Tiangen Biotech Co., Ltd., Beijing, China), and the library was constructed using the TruSeq nano DNA kit (Illumina, San Diego, CA, United States). The NextSeq 500 device (Illumina) was used for sequencing at the 75-bp single-end mode, and approximately 12 million reads were generated per sample.

### NGS data preprocessing

The generated reads were aligned with the reference human genome (hg19) using the default parameter of the BWA-MEM algorithm (v.0.7.5) (Li, 2013). The polymerase chain reaction duplicate reads were removed using Picard (v1.96; https://broadinstitute.github.io/picard/, accessed on 23 August 2014), and those showing a mapping quality below 60 were excluded from the analysis using Samtools (v1.2) (Li et al., 2009). In order to adjust the GC content and mappability, the default options of the readDepth package (v.0.9.8.4) for R were applied (Miller et al., 2011). For estimating the fetal fraction, the default options of the SeqFF algorithm were applied (Kim et al., 2015).

### Z-score, NCV-score, and CNV analyses

After adjusting the GC content and mappability, the resultant values were used to calculate the Z-score. The total sum of samples was used in the normalization of each chromosome to be analyzed. From the low-risk groups in the development set, 994 samples were randomly selected as the normal reference cohort to calculate the mean and SD for the normalized values of chromosomes 21, 18, and 13 in order to obtain the Z-score (Chiu et al., 2008), with the cutoff set at 3. In the NCV analysis, normalization was performed using the normalizing denominator suitable for each target chromosome: chromosome 9 for chromosome 21 as the target, chromosome 8 for chromosome 18 as the target, and the sum of 2–6 chromosomes for chromosome 13 as the target (Sehnert et al., 2011). Using the same reference cohort applied to the Z-score calculation, the mean and SD were calculated for the normalized values of chromosomes 21, 18, and 13, with the cutoff set at 3. The WISECONDOR (WIthin-SamplE COpy Number aberration DetectOR) method was used for the CNV analysis (Straver et al., 2014).

### Calculation of FD and TRS image generation

**FIGURE 4:**
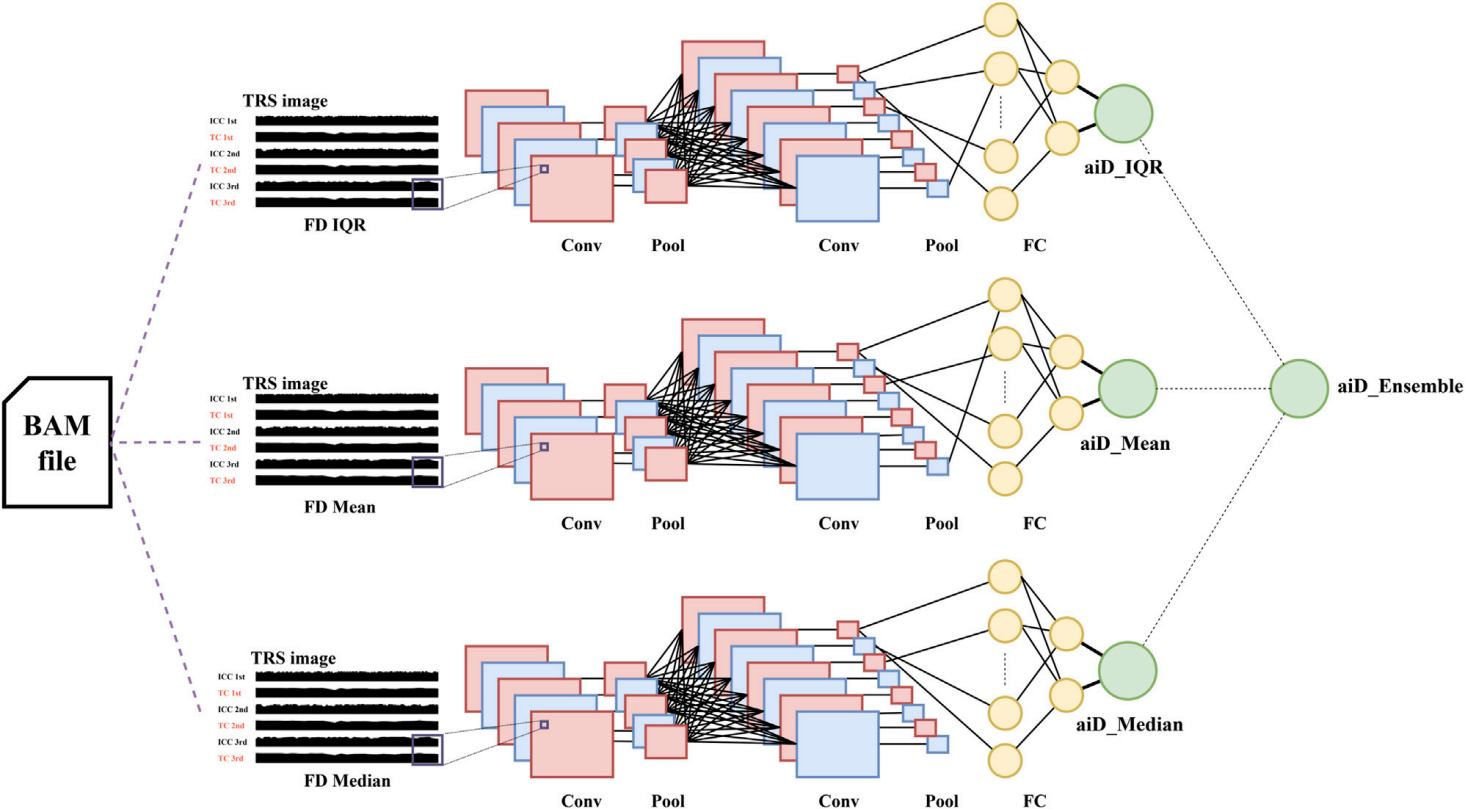
aiD-NIPT training and ensemble process. Through target repeat stacking (TRS) image generation using the fragment distance (FD) representative values (mean, median, and IQR), the respective convolutional neural network algorithms were produced. Convolutional Neural Network (CNN) consists of the convolution process and a neural network. In the convolution process, the convolution (Conv) and max pooling (Pool) processes are repeated. Thereafter, a fully connected layer (FC) is created and neural network analysis is performed. The median of the probability values was used for the ensemble. aiD: artificial intelligence of fragment distance; BAM: the binary version of a SAM file; Conv: convolution; FC: Fully Connected; ICC: internal control chromosome; NIPT: non-invasive prenatal testing; IQR: interquartile range; TC: target chromosome.

FD is defined as the difference in the aligned positions between two adjacent fragments. The data used in this study were single-end reads, and shifting was performed to accurately define the positional values of fragments. For forward reads, 80 bp was added to the minimum value of the aligned positions. For reversely aligned reads, 80 bp was subtracted from the maximum positional value (Figure 2A). TRS images were generated as the input data for the AI analysis. For TRS, the target chromosome was repeatedly stacked across the internal control chromosome (ICC) (Figure 4). For the ICC, three chromosomes with the highest similarity of the reciprocal of median chromosomal FD to the target chromosome were selected. The data of 994 normal reference samples in the development set were used to perform linear regression between the target chromosome and each autosome using the reciprocal of the median FD. Subsequently, the mean squared error (MSE) was calculated, and three chromosomes with the highest −log10 (MSE) were selected (Supplementary Table S1).

For the TRS image generation, data analysis was performed in the following steps:

Step 1 Non-overlapping binning at 1 Mbp

Step 2 Removal of problematic regions, such as centromeres and telomeres

Step 3 Removal of bins with mappability ≤80

Step 4 Removal of bins with GC content ≤30 or >50

Step 5 Calculation of FD representative values (i.e., Mean, Median and IQR) for each bin

Step 6 Definition of FD median values of each bin calculated in Step 5 as global_median

Step 7 Calculation of FD representative values in Step 5 normalized with global_median as defined in Step 6 (median normalization)

Step 8 Removal of lower 10% and upper 10% bins of the normalized FD representative values in Step 7 from the ICC and target chromosome

Step 9 Sequential line plotting of the values obtained in Step 8 for the ICC and target chromosome

The image size was set to 400 px width and 200 px length with 200 ppi resolution.

### aiD-NIPT model generation

To train the aiD-NIPT model, the development dataset was used. The composition was as follows: low-risk (*n* = 987), trisomy 21 (*n* = 162), trisomy 18 (*n* = 56), and trisomy 13 (*n* = 16). The data were divided into the training, validation, and test datasets in an approximately 5:3:2 ratio. To validate the performance of the model, five-fold cross-validation was used.

The machine learning analysis was performed using TensorFlow (v2.2.0; Google LLC) (Abadi et al., 2016). A CNN model was developed independently. TRS images used in the model training were converted to grayscale (400 × 200 × 1). The list of learning hyperparameters was as follows: learning rate, number of convolutional layers, kernel size, number of convolutional patches, number of dense layers, activation function, and dropout rate. In hyperparameter tuning for model optimization, the Bayesian optimizer was used (Snoek et al., 2012). In order to identify the model with the best hyperparameters, 200 models were constructed, and the final model was selected based on the loss value of the validation dataset. For the detection of trisomy 21, trisomy 18, and trisomy 13, each respective binary model was produced, with the positive detection cutoff set at 0.5. For the ensemble of the FD model, the median of three probability values was used. The mean of values obtained from the five-fold cross-validation was used as the final probability value. To verify the feature importance of the model, the SHAP v0.40.0 algorithm was employed (Lundberg et al., 2018), and visualization was performed by overlapping the weight value of the first layer of the model and the input image.

### Statistical analysis

Statistical analysis was performed using R (v.4.0.5). The sensitivity, specificity, PPV, and NPV of trisomy 21, trisomy 18, and trisomy 13 were calculated using the “caret” package (v.6.0-88). The 95% confidence interval was set under the assumption of standard normal distribution.).

## Data Availability

The data presented in the study are deposited in the KoNA (https://kobic.re.kr/kona) repository, accession number PRJKA220469.
