# Supplementary material for: Development and performance evaluation of an artificial intelligence algorithm using cell-free DNA fragment distance for non-invasive prenatal testing (aiD-NIPT)
Source: Front Genet. 2022 Nov 29;13:999587. doi: 10.3389/fgene.2022.999587 (PMC9745024; doi:10.3389/fgene.2022.999587)
Supplement: Supplementary file 1 [file Table1.DOCX]

Supplementary Material

# Supplementary Figures and Tables

## Supplementary Figures


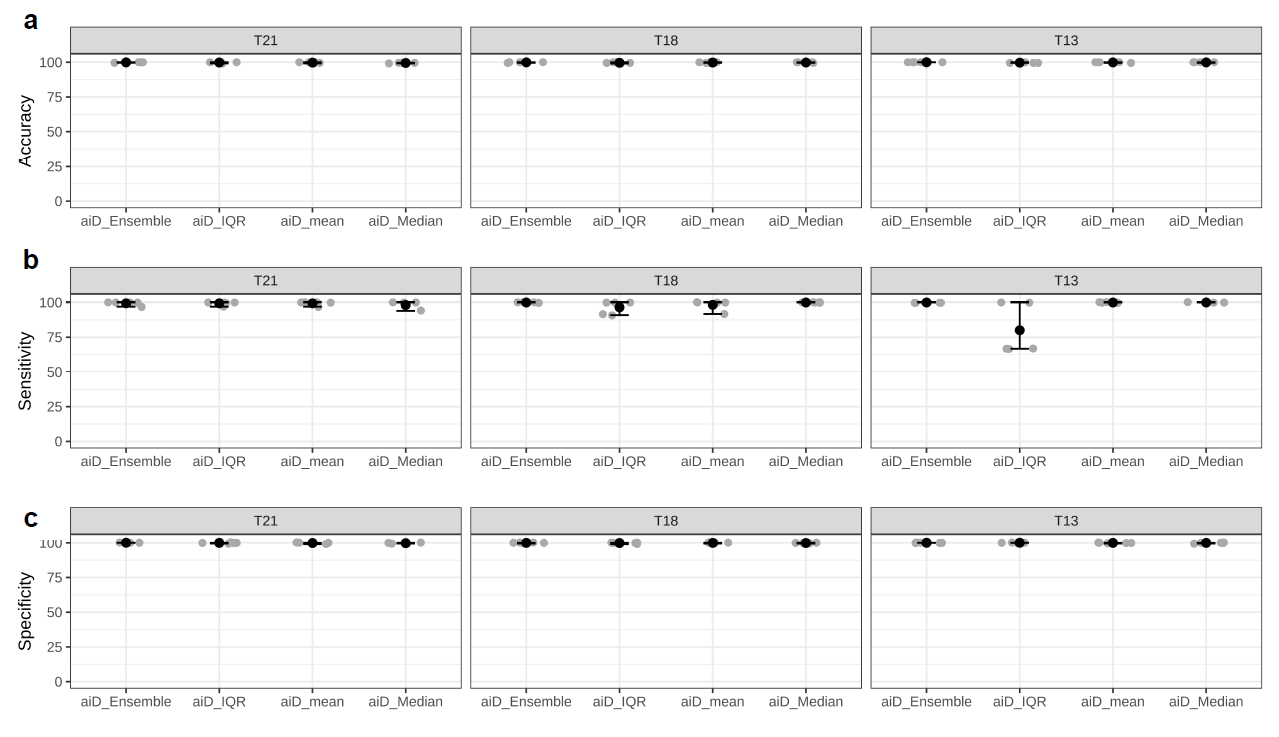


**Supplementary Figure 1**. Accuracy (a), sensitivity (b), and specificity (c) results of the aiD-NIPT algorithm for trisomy 21, trisomy 18, and trisomy 13 (five-fold cross-validation). The black dots in each image indicate the mean of the five-fold cross-validation, and the error bars indicate the minimum and maximum values from the five-fold cross-validation. aiD: artificial intelligence of fragment distance; IQR: interquartile range; NIPT: non-invasive prenatal testing.


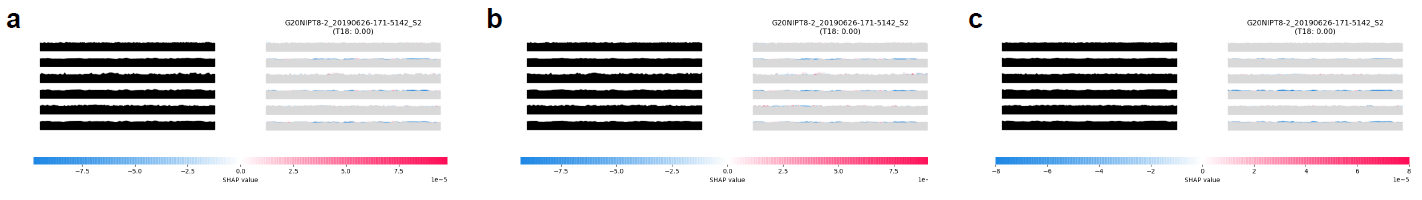


**Supplementary Figure 2.** aiD-NIPT analysis of the trisomy 18 false-negative case, presenting target repeat stacking images for aiD_IQR (a), aiD_Mean (b), and aiD_Median (c), and the distribution of SHAP values. No positive pattern was observed across all analyses. aiD: artificial intelligence of fragment distance; NIPT: non-invasive prenatal testing; IQR: interquartile range; SHAP: Shapley additive explanations

**Supplementary Table 1.** Internal control chromosome in target repeat stacking image generation

| Target chromosome | Internal control chromosome |
| --- | --- |
| 21 | 2, 12, 14 |
| 18 | 2, 7, 8 |
| 13 | 4, 5, 6 |

**Supplementary Table 2**. Results of the aiD-NIPT training and accuracy (%) of the test dataset. Mean values of the five-fold cross-validation

| Test set  accuracy (%) | aiD_Ensemble  (min–max) | aiD_IQR  (min–max) | aiD_Mean  (min–max) | aiD_Median  (min–max) |
| --- | --- | --- | --- | --- |
| Overall | 99.92  (99.59–100) | 99.51  (98.77–100) | 99.84  (99.59–100) | 99.75  (99.18–100) |
| T21 | 99.91  (99.57–100) | 99.83  (99.13–100) | 99.74  (99.13–100) | 99.48  (99.13–99.57) |
| T18 | 99.0  (99.52–100) | 99.62  (99.05–100) | 99.81  (99.52–100) | 99.81  (99.52–100) |
| T13 | 100  (100–100) | 99.70  (99.50–100) | 99.90  (99.50–100) | 99.90  (99.50–100) |

NIPT: non-invasive prenatal testing; IQR: interquartile range; aiD: artificial intelligence of fragment distance; min: minimum accuracy of five-fold cross-validation; max: maximum accuracy of five-fold cross-validation

**Supplementary Table 3.** Results of the aiD-NIPT training and sensitivity (%) of the test set. Average values from the five-fold cross-validation

| Test set  sensitivity (%) | aiD_Ensemble  (min–max) | aiD_IQR  (min–max) | aiD_Mean  (min–max) | aiD_Median  (min–max) |
| --- | --- | --- | --- | --- |
| Overall | **99.57 (99.87–100)** | **97.44 (93.62–100)** | **99.15 (97.87–100)** | **98.70 (95.65–100)** |
| T21 | **99.39 (96.97–100)** | **99.39 (96.97–100)** | **99.39 (96.97–100)** | **98.18 (93.94–100)** |
| T18 | **100 (100–100)** | **96.52 (90.91–100)** | **98.33 (91.67–100)** | **100 (100–100)** |
| T13 | **100 (100–100)** | **80.00 (66.67–100)** | **100 (100–100)** | **100 (100–100)** |

NIPT: non-invasive prenatal testing; IQR: interquartile range; aiD: artificial intelligence of fragment distance; min: minimum sensitivity of the five-fold cross-validation; max: maximum sensitivity of the five -fold cross-validation

**Supplementary Table 4.** Chromosome model-specific performance of each algorithm on the clinical dataset

| Trisomy 21  model | True positive  (n) | False negative  (n) | False positive  (n) | True negative  (n) | Sensitivity (95% CI) | Specificity (95% CI) | PPV (95% CI) | NPV (95% CI) |
| --- | --- | --- | --- | --- | --- | --- | --- | --- |
| Z-score | 84 | 1 | 81 | 17,489 | 98.82  (96.53–100) | 99.54  (99.44–99.64) | 50.91  (43.28–58.54) | 99.99  (99.98–100) |
| NCV score | 84 | 1 | 85 | 17,485 | 98.82  (96.53–100) | 99.52  (99.41–99.62) | 49.7  (42.17–57.24) | 99.99  (99.98–100) |
| aiD_Ensemble | 85 | 0 | 3 | 17,567 | 100  (100–100) | 99.98  (99.96–100) | 96.59  (92.8–100) | 100  (100–100) |
| aiD_IQR | 84 | 1 | 10 | 17,560 | 98.82  (96.53–100) | 99.94  (99.91–99.98) | 89.36  (83.13–95.59) | 99.99  (99.98–100) |
| aiD_Mean | 85 | 0 | 5 | 17,565 | 100  (100–100) | 99.97  (99.95–100) | 94.44  (89.71–99.18) | 100  (100–100) |
| aiD_Median | 84 | 1 | 22 | 17548 | 98.82  (96.53–100) | 99.87  (99.82–99.93) | 79.25  (71.52–86.97) | 99.99  (99.98–100) |
|  |  |  |  |  |  |  |  |  |
| Trisomy 18  model | **True positive**  **(n)** | **False negative**  **(n)** | **False positive**  **(n)** | **True negative**  **(n)** | **Sensitivity (95% CI)** | **Specificity (95% CI)** | **PPV (95% CI)** | **NPV (95% CI)** |
| Z-score | 18 | 1 | 36 | 17,534 | 94.74  (84.7–100) | 99.54  (99.44–99.64) | 50.91  (43.28–58.54) | 99.99  (99.98–100) |
| NCV score | 18 | 1 | 51 | 17,519 | 94.74  (84.7–100) | 99.71  (99.63–99.79) | 26.09  (15.73–36.45) | 99.99  (99.98–100) |
| aiD_Ensemble | 18 | 1 | 9 | 17,561 | 94.74  (84.7–100) | 99.95  (99.92–99.98) | 66.67  (48.89–84.45) | 99.99  (99.98–100) |
| aiD_IQR | 18 | 1 | 16 | 17,554 | 94.74  (84.7–100) | 99.91  (99.86–99.95) | 52.94  (36.16–69.72) | 99.99  (99.98–100) |
| aiD_Mean | 18 | 1 | 4 | 17,566 | 94.74  (84.7–100) | 99.98  (99.95–100) | 81.82  (65.7–97.94) | 99.99  (99.98–100) |
| aiD_Median | 17 | 2 | 25 | 17,545 | 89.47  (75.67–100) | 99.86  (99.8–99.91) | 40.48  (25.63–55.32) | 99.99  (99.97–100) |
|  |  |  |  |  |  |  |  |  |
| Trisomy 13  model | **True positive**  **(n)** | **False negative**  **(n)** | **False positive**  **(n)** | **True negative**  **(n)** | **Sensitivity (95% CI)** | **Specificity (95% CI)** | **PPV (95% CI)** | **NPV (95% CI)** |
| Z-score | 4 | 0 | 40 | 17,530 | 100  (100–100) | 99.77  (99.7–99.84) | 9.09  (0.6–17.59) | 100  (100–100) |
| NCV score | 4 | 0 | 59 | 17,511 | 100  (100–100) | 99.66  (99.58–99.75) | 6.35  (0.33–12.37) | 100  (100–100) |
| aiD_Ensemble | 4 | 0 | 2 | 17,568 | 100  (100–100) | 99.99  (99.97–100) | 66.67  (28.95–100) | 100  (100–100) |
| aiD_IQR | 4 | 0 | 2 | 17,568 | 100  (100–100) | 99.99  (99.97–100) | 66.67  (28.95–100) | 100  (100–100) |
| aiD_Mean | 4 | 0 | 6 | 17,564 | 100  (100–100) | 99.97  (99.94–99.99) | 40  (9.64–70.36) | 100  (100–100) |
| aiD_Median | 4 | 0 | 6 | 17,564 | 100  (100–100) | 99.97  (99.94–99.99) | 40  (9.64–70.36) | 100  (100–100) |

CI: confidence interval; PPV: positive predictive value; NCV: normalized chromosomal value; aiD: artificial intelligence of fragment distance; IQR: interquartile range
